# Frequency of mitochondrial m.1555A > G mutation in Syrian patients with non-syndromic hearing impairment

**DOI:** 10.1186/s12901-018-0055-2

**Published:** 2018-05-21

**Authors:** Hazem Kaheel, Andreas Breß, Mohamed A. Hassan, Aftab Ali Shah, Mutaz Amin, Yousuf H. Y. Bakhit, Marlies Kniper

**Affiliations:** 1 University, HNO –universities Klink-Tubingen, Tubingen, Germany; 2 Department of Bioinformatics, Africa city of technology, Khartoum, Sudan; 3 Faculty of Biotechnology, University of Malakand, Khyber Pakhtunkhwa, Pakistan; 4 Department of Biochemistry, Faculty of Medicine, University of Khartoum, P. O. Box 102, Khartoum, Sudan; 5 Department of Basic Medical Sciences, Faculty of Dentistry-University of Khartoum, Khartoum, Sudan; 6 Division of Molecular Genetics, Institute of Human Genetics, University of Tübingen, Tübingen, Germany, African city of Technology, Khartoum, Sudan

**Keywords:** m.1555A > G, Non-syndromic, Hearing impairment, Syria, mtDNA

## Abstract

**Background:**

Mitochondrial maternally inherited hearing impairment (HI) appears to be increasing in frequency. The incidence of mitochondrial defects causing HI is estimated to be between 6 and 33% of all hearing deficiencies. Mitochondrial m.1555A > G mutation is the first mtDNA mutation associated with non-syndromic sensorineural deafness and also with aminoglycoside induced HI. Its prevalence varied geographically between different populations.

**Methods:**

We carried out PCR, restriction enzyme based screening, and sequencing of 337 subjects (including 132 patients diagnosed clinically with hereditary deafness) from 54 families from Syria for m.1555A > G mitochondrial mutation.

**Results:**

Mitochondrial m.1555A > G mutation was detected in one of fifty-four families (1.85%), six out of the 132 (4.5%) of all patients with NSHI and one propositus of the 205 individuals with normal hearing (0.48%).

**Conclusion:**

This is the first study to report prelingual deafness causative gene mutations identified by sequencing technology in Syrian families. It is obvious from the results that the testing for the m.1555A > G mutation is useful for diagnosis of hearing loss in Syrian patients and should also be considered prior to treatment with aminoglycosides in predisposed individuals.

## Background

Hearing impairments (HI) is as one of the most disabling disorders in the world, limiting a person’s ability to communicate with others [1]. It is caused by genetic and/or environmental factors. Genetically caused HI is 70% non-syndromic and 30% syndromic [2], with each form related to specific genes. Non-syndromic hearing impairment (NSHI) is isolated hearing deficit without other medical derangements [3]. It affects about 0.1% of live newborns and 4% of all people below 45 years of age [4]. This number increases dramatically in countries and regions where consanguineous marriages are common, like Syria and other Middle Eastern countries [5–7]. Up to 75–80% of cases of non-syndromic hearing impairment have an autosomal recessive cause, 10–15% inherited as autosomal dominant, and few are X-linked or mitochondrial [8].

Mitochondria are intracellular organelles that contain their own DNA (mtDNA). A substitution of A- > G nitrogenous bases in position 1555 of the mitochondrial *12S rRNA* gene is the most prevalent mutation in the mitochondrial genome, associated with both late onset and congenital NSHI. It is of particular interest being a key cause of antibiotic-induced HI [9]. This mutation occurs in a highly conserved region of the *12S rRNA* gene, where aminoglycosides are known to bind and result in defective ATP production in cochlear cells [10].

The m.1555A > G mutation has been identified in more than 120 families throughout the world with frequencies differing according to the population’s ethnic group [11–14]. So far, no report exists regarding the involvement of the mtDNA mutations in pathogenesis of deafness in Syria. Syria is a country in the Middle East, along the eastern shore of the Mediterranean Sea. The purpose of this study was to determine the prevalence of m.1555A > G mitochondrial mutation among patients with NSHI from Syria.

## Methods

All subjects were recruited from Aleppo, which lies in the northwestern region of Syria, with the help of deafness related schools and institutes. The examined group consisted of 337 subjects including 132 affected patients from 54 Syrian families with 2 or more affected subjects (up to 6) with prelingual, profound, sensorineural, bilateral, non-syndromic hearing loss and 205 unrelated healthy controls.

### Patients

In this study 175 females and 162 males were participated, with an age range between two and 72 years (mean age 18.2 years). Detailed family pedigrees were drawn. Information on consanguinity, age at onset of HL, detailed prenatal and perinatal history, use of ototoxic drugs (aminoglycosides), etc. was obtained through a detailed questionnaire. Physical examination was performed to exclude syndromic forms of hearing impairment at Alrazi Hospital (Aleppo), ENT examination such as Tympanometry, and BERA (Brainstem Electric Response Audiometry) were also applied at ENT department of the hospital Alrazi.

### Mutation Screening

**Fig. 1:**
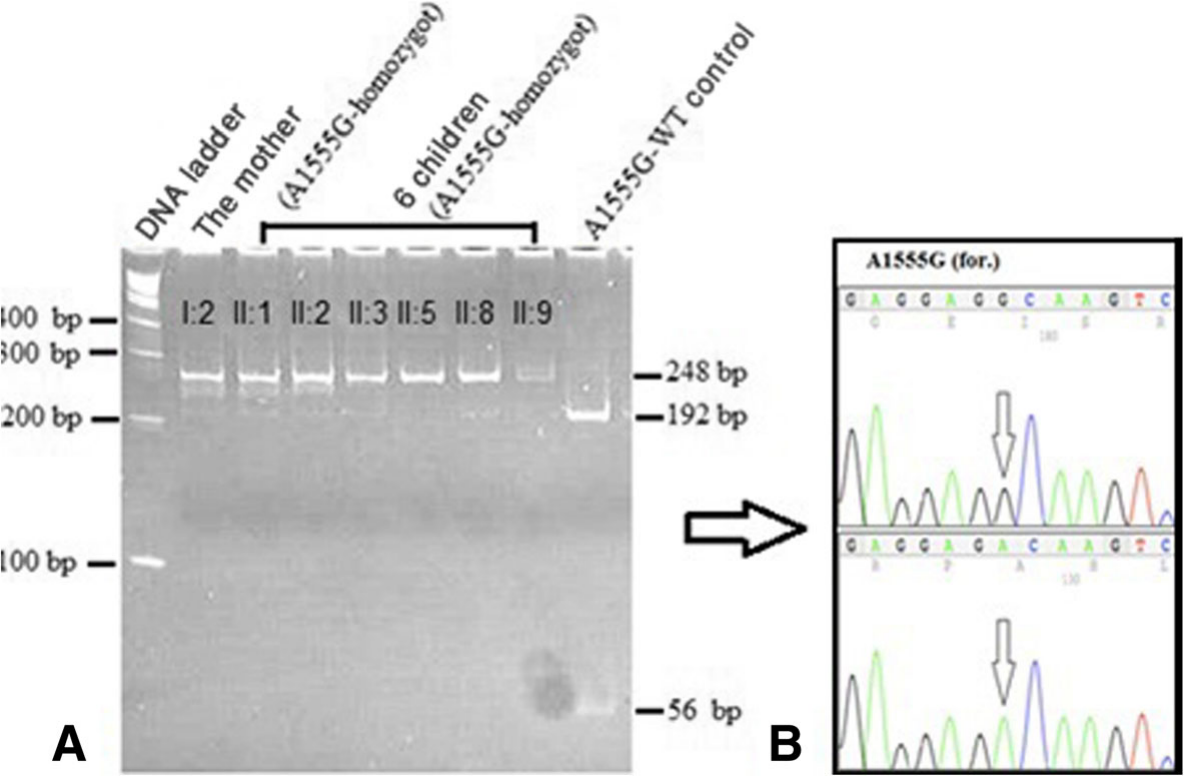
(**a**): PCR-RFLP analysis of the only Syrian family found with m.1555A > G mutation: a 248 bp PCR fragment is digested with BsmAI. DNA ladder (the first panel). The wild-type mtDNA is cleaved in to tow fragments, 192 and 56 bp in length (the last panel). PCR product containing the m.1555A > G mutation is not cleaved (the other left panels). (**b**): Partial Sequence chromatograms from a normal hearing individual (down) and affected proband with the m.1555A > G mutation in the mitochondrial *12S rRNA* gene (top) with the forward and reverse primers. The small arrows indicate the localization of the change of an Adenine to Guanine nucleotide at position 1555 of the *12S rRNA* gene

10 ml of peripheral venous blood was obtained from each affected and healthy family members. Genomic DNA was extracted using Qiagen FlexiGene kits in accordance with the manufacturer’s instructions. Mitochondrial DNA m.1555A > G mutation was detected using PCR-RFLP strategy (m.1555A > G, F: AGAAATGGGCTACATTTTCTACCC; m.1555A > G, R: GTTCGTCCAAGTGCACCTTCCA) as mentioned in [15] followed by BsmAI-digest (site: GTC TCN/) (New England Bio Labs®). Products with m.1555A > G mutation showed one band with 248 bp and wild individual displayed two bands (192 bp and 56 bp) after polyacrylamide gel electrophoresis (6%)(Fig. 1a).

The presence of the m.1555A > G mutation was verified using standard Sanger sequencing. Detected mutation was confirmed at least two times and compared with the reference sequence (NC_012920).

## Results

The m.1555A > G mutation was found in only one out of 54 families with NSHI(Fig. 1a). The family consisted of four generations. The m.1555A > G mutation was detected in six out of the 132 NSHI patients and in one of the 205 individuals with normal hearing. This result was confirmed by direct sequencing of the corresponding PCR product (Fig. 1b).

**Fig. 2:**
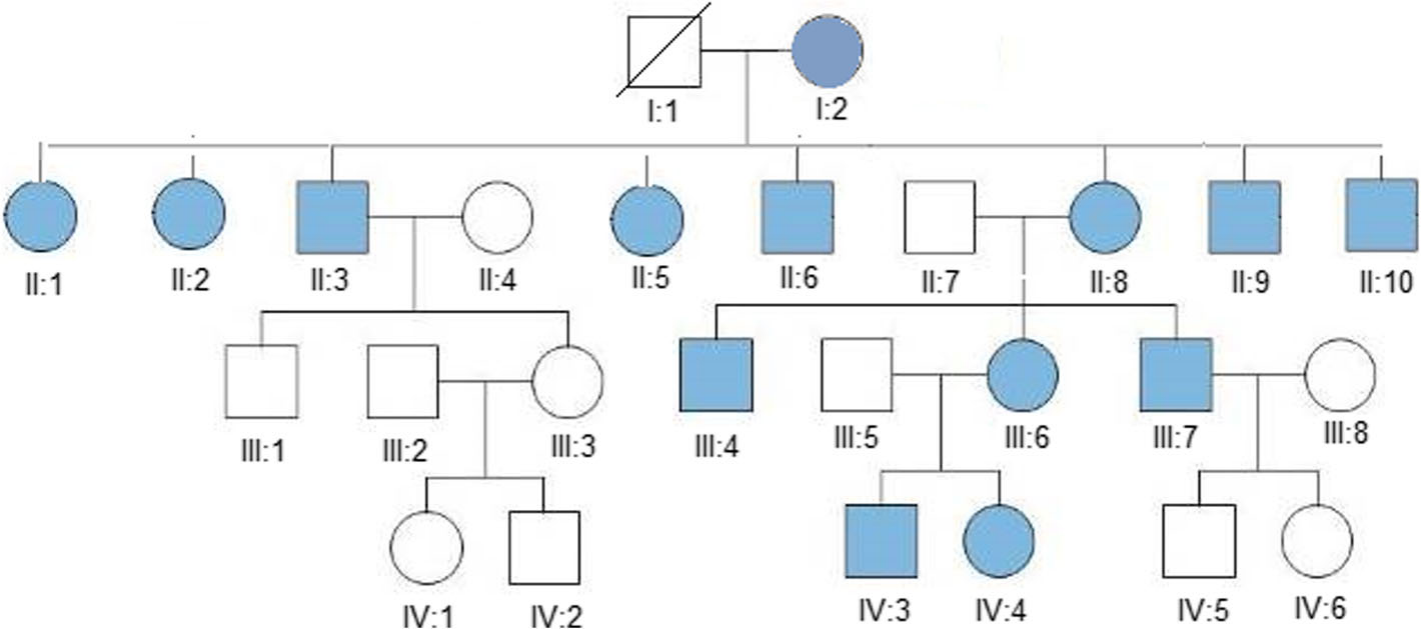
A graphic map of a four generation large pedigree of a Syrian family found with m.1555A > G mutation in our study. The squares represent males, while the circles represent females. Blue filled shapes are affected individuals (deaf), white filled shapes are unaffected (controls)

The family pedigree of patients with m.1555A > G mutation is shown in Fig. 2. Proposita (I: 2, Fig. 2.) is a 72 year old female with age related hearing impairment (presbycusis). She had eight offspring (4 males and 4 females) all with congenital, profound, bilateral and sensorineural deafness since childhood in accordance with mitochondrial inheritance (Fig. 2). Unfortunately drug history of aminoglycoside usage in this family was unavailable. The m.1555A > G mutation in the mitochondrial *12S rRNA* gene was found in this mother and all her investigated offspring (Fig. 2).

## Discussion

The mtDNA m.1555A > G mutation is the most prevalent mutation of mitochondrial genome, associated with both late onset and congenital NSHI, with aminoglycoside-induced hearing loss, in people with different ethnic backgrounds [16]. This is the first study to report the prevalence of m.1555A > G mitochondrial mutation in Syria, one of the Middle East countries. We found the m.1555A > G mutation in one out fifty-four families (1.85%), six out of the 132 NSHI patients (4.5%) and one proposita of the 205 individuals with a normal hearing (0.48%). It shows that the average frequency of the m.1555A > G mutation is 1.85% in the Syrian deaf population. Similar results were previously found in Egypt [17] and reports from other Caucasian populations [7, 12, 18], but not other Middle Eastern countries like Qatar [19], and Iran [20] probably due to the ethnic differences between these populations and the population under study.

Even though it has now been clearly shown that the Prevalence this mutation (1.85%) is not so high in familial cases in Syria, it has nevertheless a very important role. Detection of this mutation would delay the onset of the disease or even prevent it through a lifelong strict avoidance of taking aminoglycosides. When indicated, such a genetic analysis prior to the administration of aminoglycosides would be very valuable, since even a single, small parenteral administration may cause bilateral deafness as a serious side effect.. These findings will help the establishment of effective diagnosis for nonsyndromic hearing loss, improve genetic counselling, and serve as a potential therapeutic platform in the future for the affected patients in Syria.

## Conclusion

This is the first study to report prelingual deafness causative gene mutations identified by sequencing technology in Syrian families. It is obvious from the results that the testing for the m.1555A > G mutation is useful for diagnosis of hearing loss in Syrian patients and should also be considered prior to treatment with aminoglycosides in predisposed individuals.

## Abbreviations

ATP: Adenosine Tri Phosphate
ENT: Ear Nose Throat
HI: Hearing Impairement
mDNA: mitochondrial Deoxy-ribonucleic Acid
NSHI: Non-syndromic Hearing Impairment
PCR: Polymerase Chain Reaction
